# Association between aldosterone and the risk of cardiovascular disease in hypertensive patients: a cohort study

**DOI:** 10.3389/fendo.2026.1791569

**Published:** 2026-04-23

**Authors:** Lin Gan, Nanfang Li, Mulalibieke Heizhati, Hui Wang, Ling Yao, Li Cai, Shasha Liu, Jing Hong, Delian Zhang

**Affiliations:** 1 Graduate School, Xinjiang Medical University, Urumqi, China; 2 Hypertension Center of People’s Hospital of Xinjiang Uygur Autonomous Region, Xinjiang Hypertension Institute, NHC Key Laboratory of Hypertension Clinical Research, Key Laboratory of Xinjiang Uygur Autonomous Region “Hypertension Research Laboratory”, Xinjiang Clinical Medical Research Center for Hypertension (Cardio-Cerebrovascular) Diseases, Urumqi, China

**Keywords:** Aldosterone, cardiovascular disease, cohort study, hypertension, primary aldosteronism

## Abstract

**Objective:**

Whether plasma aldosterone concentration (PAC) is associated with incident cardiovascular disease (CVD) remains unclear in hypertensive patients, particularly after excluding those with primary aldosteronism. This study evaluates the association between PAC and incident CVD.

**Methods:**

This study included hypertensive patients admitted to the hypertension center between January 1, 2014, and December 31, 2017, who had PAC measurements. Incident events encompassed both cardiac events and stroke. Cox proportional hazards models were used to evaluate the association between PAC and the outcome.

**Results:**

A total of 8653 hypertensive patients were included in this study. During a median follow-up of 5.2 years, 737 cases of incident CVD occurred. The incidence of the outcome increased with higher quartiles of PAC. Individuals in the highest quartile of PAC had a 51% increased risk of CVD (HR 1.51, 95% CI 1.23–1.86) compared to those in the lowest quartile. The overall results remained stable and consistent in the stratification and sensitivity analyses.

**Conclusions:**

Higher PAC was associated with increased risk of CVD in hypertensive patients, irrespective of concomitant primary aldosteronism. These findings suggest that PAC might be a target for early prevention.

## 1 Introduction

Aldosterone, a steroid hormone secreted by the adrenal cortex, binds to the mineralocorticoid receptor (MR) in distal renal tubule epithelial cells and performs essential physiological functions such as regulating electrolyte balance, blood pressure, and blood volume (1). MRs are also expressed in the heart, liver, brain, blood vessels, and adipose tissue (2, 3). Excessive aldosterone production activates these MRs, which promotes adverse cardiorenal remodeling and vascular damage by inducing inflammation, endothelial dysfunction, and fibrosis (4).

Primary aldosteronism (PA), a condition marked by autonomous aldosterone production and suppressed plasma renin activity, is a common and frequently underdiagnosed form of secondary hypertension (5, 6). Patients with PA face a significantly elevated risk of cardiovascular morbidity and mortality compared to those with essential hypertension (7). This increased risk can be mitigated by adrenalectomy in cases of unilateral PA or through medical therapy with MR antagonists for bilateral forms (8–10). Moreover, recent studies indicate that even mild or subclinical PA is associated with inappropriate MR activation, elevated blood pressure, and adverse cardiovascular outcomes (11–14).

The association between aldosterone and cardiovascular damage is well-established in animal studies (15, 16), yet human studies have yielded conflicting results. In the Jackson Heart Study, elevated aldosterone and plasma renin activity correlated with a higher risk of CVD and all-cause mortality among community-dwelling African Americans (17). Conversely, the Framingham Offspring Study found no association between aldosterone levels and CVD in the general population (18). The relationships between aldosterone and long-term adverse cardiovascular outcomes also vary considerably among patients with high-risk CVD, including obstructive sleep apnea, heart failure, acute myocardial infarction, and coronary artery disease (19–24). Recent evidence further suggests that aldosterone is linked to all-cause mortality (25) or adverse cardiac remodeling (26) only when renin activity is suppressed (≤0.5 ng/mL per hour). These discrepancies may be partly attributable to differences in disease state, ethnicity, or the potential influence of PA. Despite these inconsistent findings, no large-scale study has specifically examined the relationship between aldosterone and CVD in hypertensive patients after systematically excluding PA. Understanding this relationship is critical for determining whether PAC should be routinely measured for cardiovascular risk stratification in hypertension.

In the present study, we used data from a large hypertension center to investigate the longitudinal association between plasma aldosterone concentrations (PAC) and incident CVD. We hypothesized that higher PAC would be associated with an increased risk of CVD, independent of PA.

## 2 Materials and methods

### 2.1 Study population

We retrospectively identified hypertensive patients admitted to the Hypertension Center of People’s Hospital of Xinjiang Uygur Autonomous Region between January 1, 2014 and December 31, 2017, whose PAC levels were measured at admission. Data were extracted from the electronic medical record system. We excluded patients with a confirmed diagnosis of PA (n=1385) or other forms of secondary hypertension, including renovascular hypertension, renal parenchymal hypertension, Cushing’s syndrome, and pheochromocytoma (n=387). The diagnostic criteria for secondary hypertension are detailed in the Supplementary Material. Additional exclusions comprised patients with stage 3–5 chronic kidney disease (n=348), and those with liver enzyme elevations exceeding three times the upper limit of normal (n=97). We also excluded individuals with a history of CVD, including myocardial infarction, heart failure, stroke, unstable angina, coronary revascularization, and coronary bypass surgery (n=553), as well as patients lost to follow-up (n=1315).

**Figure 1:**
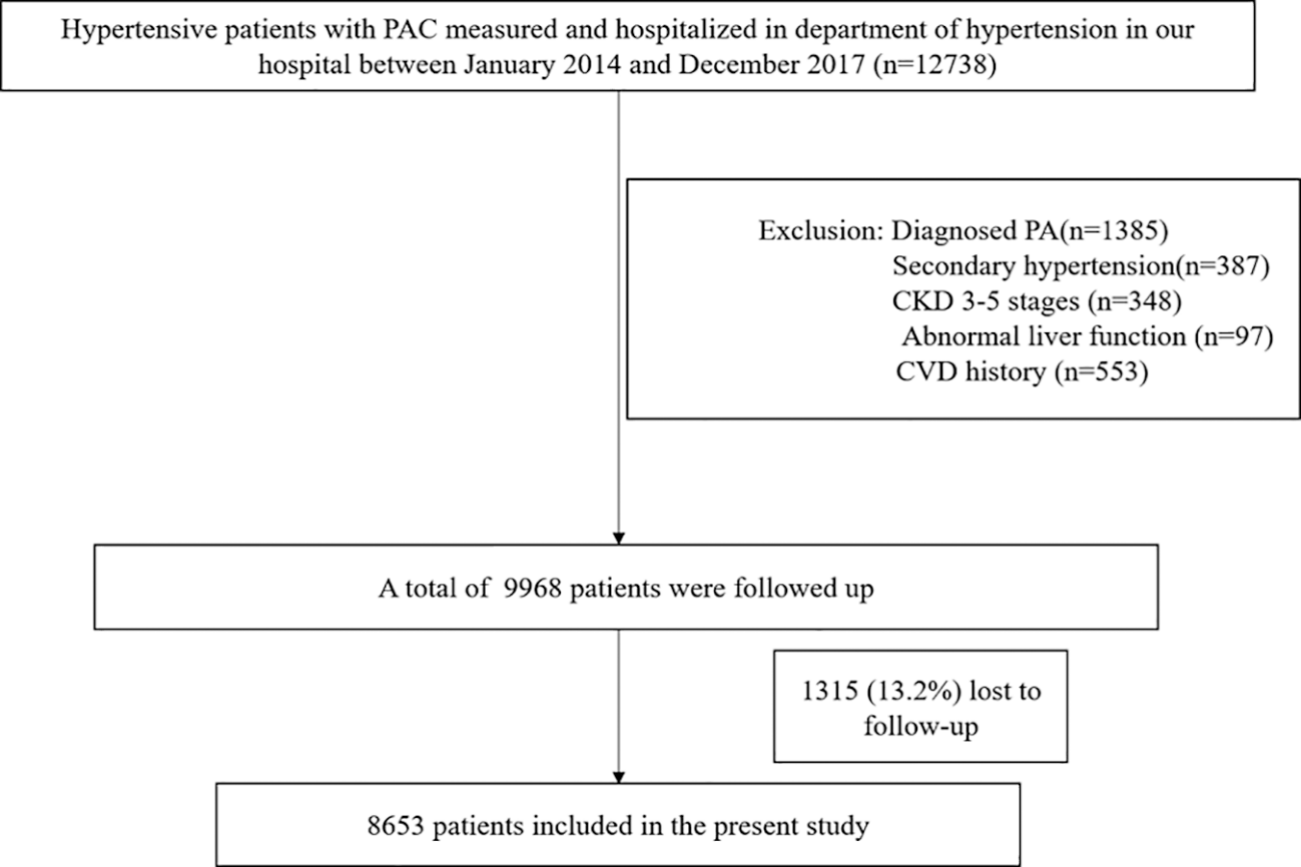
Flowchart of participants for the present study. CKD, chronic kidney disease; PA, primary aldosteronism; PAC, plasma aldosterone concentration.

The final analytical sample comprised 8653 individuals (**Figure 1**). The study protocol received approval from the Ethics Committee of the People’s Hospital of Xinjiang Uygur Autonomous Region. All eligible participants provided written informed consent.

### 2.2 Measurements of plasma aldosterone concentration, plasma renin activity

Measurements of PAC and PRA in our center have been described previously (27). Briefly, fasting blood samples were collected between 08:00 am and 11:00 am after patients had been ambulant for at least 2 hours and seated for 30 minutes. Patients were initially screened for primary aldosteronism under standardized conditions, which required the withdrawal of interfering medications for 4–6 weeks. Patients with a blood pressure of ≥160/100 mmHg were transitioned to alpha-blockers and/or calcium channel blockers like verapamil. Oral potassium supplements were administered to correct hypokalemia, targeting a serum potassium level between 3.9 and 4.0 mmol/L, while all patients maintained a full diet with normal salt intake. Suspected cases, defined by a PAC ≥12 ng/dL and an aldosterone-to-renin ratio ≥20, subsequently underwent a confirmatory saline infusion test (SIT). The diagnosis of PA was based on SIT criteria following the Endocrine Society Guideline (28). Post-SIT PAC >10 ng/dL confirmed as primary aldosteronism. Among the 12738 patients, 3702 met the screening criteria for PA (ARR≥20 and PAC≥12). Subsequently, 1955 of these patients underwent confirmatory SIT, which confirmed PA in 1385 cases. Within the final analysis cohort of 8653 individuals, 1547 patients who did not complete SIT were included in sensitivity analyses under the designation ‘suspected PA’. PAC was measured using radioimmunoassay (DSL-8600 ACTIVE Aldosterone Coated Tube Radioimmunoassay Kit; Diagnostic Systems Laboratories, Webster, TX, USA). PRA was measured by radioimmunoassay using commercial kits (Center of Beifang Biology Technique, Beijing, China).

### 2.3 Data collection

Baseline data were obtained from the medical electronic system and included age, gender, body mass index (BMI), waist circumference, smoking status, alcohol use, duration of hypertension, diabetes mellitus (T2DM), systolic blood pressure (SBP), diastolic blood pressure (DBP), uric acid (UA), serum creatinine (Scr), blood urea nitrogen (BUN), fasting blood glucose (FBG), total cholesterol (TC), triglycerides (TG), high density lipoprotein cholesterol (HDL-C), low density lipoprotein cholesterol (LDL-C), lipoprotein a (Lp a), homocysteine, serum potassium, serum sodium, 24-hour urinary potassium (24-h UK), and 24-hour urinary sodium (24-h UNa). We also recorded discharge medications, such as antihypertensive agents, statins, antiplatelet agents, and antidiabetic drugs. The estimated glomerular filtration rate (eGFR) was calculated using the CKD-EPI equation.

### 2.4 Follow up and outcomes

The primary outcome was new-onset CVD, which included non-fatal stroke–comprising ischemic stroke (ICD-10: I63) and hemorrhagic stroke (ICD-10: I60- I61)–and cardiac events, such as acute myocardial infarction (ICD-10: I21-I22), coronary revascularization, hospitalized unstable angina, and hospitalized heart failure. Coronary revascularization was defined as either percutaneous coronary intervention with stent implantation or coronary artery bypass grafting. Hospitalization for heart failure or unstable angina was defined as any unplanned inpatient stay, lasting one or more nights, in a hospital or equivalent facility where the primary admission diagnosis was heart failure or unstable angina. Outcome diagnoses during follow-up were primarily obtained from medical records and the Xinjiang social medical insurance system. All outcomes were independently verified by two investigators to ensure accuracy. The investigators were blinded to the PAC levels. The follow-up period concluded on 30 April 2023.

### 2.5 Statistical analyses

Continuous variables with a normal distribution are reported as mean ± standard deviation (SD) and were compared using analysis of variance (ANOVA). Non-normally distributed continuous variables are presented as median (interquartile range) and were compared with the Kruskal-Wallis H test. Categorical variables were expressed as frequency and percentage, and group comparisons were performed using the chi-square test. Missing data constituted less than 1% of any variable, and means were used to impute these missing values.

The association between PAC and CVD, cardiac events and stroke was assessed using Kaplan-Meier curves, with comparisons made via the log-rank test. Cox proportional hazards regression models estimated the multivariable-adjusted risk of outcomes, treating PAC as either categorical (quartiles) or continuous variables (per SD increase). Multicollinearity was evaluated for all variables in the multiple Cox regression models, with a variance inflation factor >10 or tolerance <0.10 indicating potential concern. In this test, both TC and LDL-C exhibited VIF>10; TC was therefore excluded from subsequent analysis (**Supplementary Table 1**). Three multivariate models were constructed based on univariate Cox regression results and clinical plausibility: Model 1 adjusted for age, gender, hypertension duration ≥5 years, smoking status, and alcohol use; Model 2 included Model 1 covariates plus BMI, waist circumference, SBP, DBP, eGFR, BUN, TG, HDL-C, LDL-C, Lp(a), FBG, 24-h UNa, and 24-h UK; Model 3 further incorporated discharge medications, including antihypertensive agents, statins, antiplatelet agents, and antidiabetic drugs. Restricted cubic spline (RCS) was performed to examine the shape of the association between PAC and CVD with 3 knots (at the 10th, 50th, 90th percentiles).

Stratification analyses further assessed the associations between PAC and CVD across age, gender, BMI, smoking status, alcohol use, baseline T2D, SBP, DBP, 24-h UNa, 24-h UK, and PRA. To verify the robustness of the findings, sensitivity analyses excluded patients who developed CVD within one year or those with suspected PA (defined as ARR ≥ 20 and PAC ≥ 12). Statistical significance was defined as a two-sided P < 0.05. All analyses were conducted using R version 4.2.2 and SPSS version 25.0 for Windows.

## 3 Results

### 3.1 Baseline characteristics

This study included 8653 participants with a mean age of 50.4 (SD 11.8) years; 53.7% were men, 22.9% were cigarette consumers, and 24.2% were alcohol users. Mean SBP and DBP were 146.9 ± 20.8mmhg and 89.3 ± 15.1mmhg, respectively.

Participants in the highest PAC quartile tended to be younger, and exhibited higher SBP, DBP, eGFR, LDL-C, 24-h UK, and PRA. They also demonstrated lower serum potassium levels. The baseline characteristics of participants across PAC quartiles are detailed in **Table 1**.

**Table 1:** Baseline characteristics of groups classified by PAC quartiles.

	Overall	Quartile 1	Quartile 2	Quartile 3	Quartile 4	P value
	(n=8653)	<11.84(n=2157)	11.84-14.21(n=2166)	14.22-19.73(n=2166)	>19.73(n=2164)	
Gender, male (%)	4649(53.7)	1101(51.0)	1153(53.2)	1229(56.7)	1166(53.9)	0.002
Age (year)	50.4 ± 11.8	51.4 ± 12.2	50.6 ± 11.6	50.5 ± 11.6	49.3 ± 11.9	<0.001
BMI (kg/m^2^)	27.1 ± 3.9	27.1 ± 3.9	27.1 ± 3.8	27.3 ± 3.9	27.1 ± 3.9	0.278
Waist circumference (cm)	97.7 ± 11.3	97.9 ± 11.4	97.7 ± 11.2	97.9 ± 11.4	97.3 ± 11.1	0.210
Smoking status (yes, n%)	1979(22.9)	461(21.4)	501(23.1)	539(24.9)	478(22.1)	0.036
Alcohol use (yes, n%)	2095(24.2)	473(21.9)	529(24.4)	568(26.2)	525(24.3)	0.012
Hypertension Duration (≥5 years, n%)	4078(47.1)	991(45.9)	1030(47.6)	1024(47.3)	1033(47.7)	0.634
DM at baseline (%)	1392(16.1)	383(17.8)	353(16.3)	314(14.5)	342(15.8)	0.033
Systolic blood pressure (mmHg)	146.9 ± 20.8	145.4 ± 20.4	146.5 ± 20.4	146.5 ± 20.2	149.4 ± 21.8	<0.001
Diastolic blood pressure (mmHg)	89.3 ± 15.1	87.3 ± 14.3	88.0 ± 14.5	89.7 ± 14.6	92.5 ± 16.2	<0.001
eGFR (ml/min per 1.73m^2^)	100.3 ± 13.8	99.6 ± 13.7	99.9 ± 13.3	100.7 ± 13.7	101.0 ± 14.5	0.002
Blood urea nitrogen (mmol/L)	5.11 ± 1.36	5.13 ± 1.31	5.11 ± 1.39	5.11 ± 1.34	5.10 ± 1.40	0.946
Uric acid (μmol/L)	334.5 ± 92.4	321.2 ± 90.3	333.1 ± 89.8	341.3 ± 94.0	342.1 ± 94.0	<0.001
Alanine aminotransferase (U/L)	25.6 ± 16.7	24.3 ± 16.2	25.3 ± 16.1	26.2 ± 17.0	26.7 ± 17.4	<0.001
Aspartate aminotransferase (U/L)	20.2 ± 8.5	20.0 ± 8.4	20.1 ± 8.4	20.2 ± 8.0	20.6 ± 9.0	0.115
Fasting blood glucose (mmol/L)	5.09 ± 1.69	5.17 ± 1.72	5.06 ± 1.51	5.02 ± 1.54	5.13 ± 1.95	0.020
Total cholesterol (mmol/L)	4.48 ± 0.96	4.44 ± 0.96	4.51 ± 1.00	4.47 ± 0.93	4.51 ± 0.94	0.044
Triglyceride (mmol/L)	1.90 ± 1.42	1.84 ± 1.49	1.89 ± 1.37	1.96 ± 1.48	1.91 ± 1.33	0.047
HDL-C (mmol/L)	1.02 ± 0.24	1.02 ± 0.24	1.03 ± 0.25	1.01 ± 0.23	1.02 ± 0.24	0.038
LDL-C (mmol/L)	2.70 ± 0.79	2.65 ± 0.78	2.71 ± 0.80	2.69 ± 0.79	2.73 ± 0.80	0.004
Lp (a) (mg/L)	107.8(54.2,194.4)	112.2(61.2,199.6)	104.6(48.9,194.6)	106.5(52.0,197.3)	107.3(52.9,188.4)	0.005
Homocysteine (μmol/L)	14.8 ± 6.8	15.6 ± 7.6	14.1 ± 6.3	14.6 ± 6.3	15.0 ± 7.0	<0.001
Serum potassium (mmol/L)	3.85 ± 0.37	3.86 ± 0.36	3.87 ± 0.37	3.86 ± 0.37	3.80 ± 0.39	<0.001
Serum sodium (mmol/L)	141.5 ± 2.5	141.6 ± 2.7	141.7 ± 2.4	141.5 ± 2.4	141.3 ± 2.5	<0.001
24-h UNa (mmol/L)	156.1 ± 73.4	159.5 ± 71.3	157.1 ± 74.4	155.9 ± 71.3	152.1 ± 76.4	0.010
24-h UK (mmol/L)	36.2 ± 14.1	34.4 ± 12.9	35.5 ± 15.0	36.8 ± 13.4	37.9 ± 14.7	<0.001
PRA (ng/ml per h)	1.75(0.71,3.07)	1.17(0.48,2.41)	1.42(0.60,2.46)	2.01(0.86,3.17)	2.39(1.35,3.64)	<0.001
ARR (ng/dl)/(ng/ml per h)	9.10(5.32,19.67)	8.07(3.89,19.74)	9.09(5.27,21.07)	8.72(5.30,19.04)	10.09(6.72,18.65)	<0.001
Medications at discharge						
ACEI/ARB (%)	4595(53.1)	1222(56.7)	1127(52.0)	1131(52.2)	1115(51.5)	0.002
β-blocker (%)	1262(14.6)	362(16.8)	322(14.9)	260(12.0)	318(14.7)	<0.001
CCB (%)	5679(65.6)	1417(65.7)	1375(63.5)	1418(65.5)	1469(67.9)	0.025
Diuretic (%)	753(8.7)	212(9.8)	176(8.1)	176(8.1)	189(8.7)	0.154
Antihypertensive agents≥2	4939(57.1)	1224(56.7)	1204(55.6)	1198(55.3)	1313(60.7)	0.001
Statins (%)	6025(69.6)	1589(73.7)	1509(69.7)	1485(68.6)	1442(66.6)	<0.001
Antiplatelet agents (%)	4638(53.6)	1238(57.4)	1155(53.3)	1154(53.3)	1091(50.4)	<0.001
Antidiabetic drugs (%)	1300(15.0)	354(16.4)	329(15.2)	289(13.3)	328(15.2)	0.043

ACEI, angiotensin-converting-enzyme inhibitors; ARB, angiotensin receptor blockers; ARR, aldosterone to renin activity ratio; BMI, body mass index; CCB, calcium channel blockers; eGFR, estimated glomerular filtration rate; HDL-C, high density lipoprotein cholesterol; LDL-C, low density lipoprotein cholesterol; PRA, plasma renin activity; 24-h UNa, 24-h urinary sodium excretion; 24-h UK, 24-h urinary potassium excretion.

### 3.2 Association of aldosterone with cardiovascular disease

**Figure 2:**
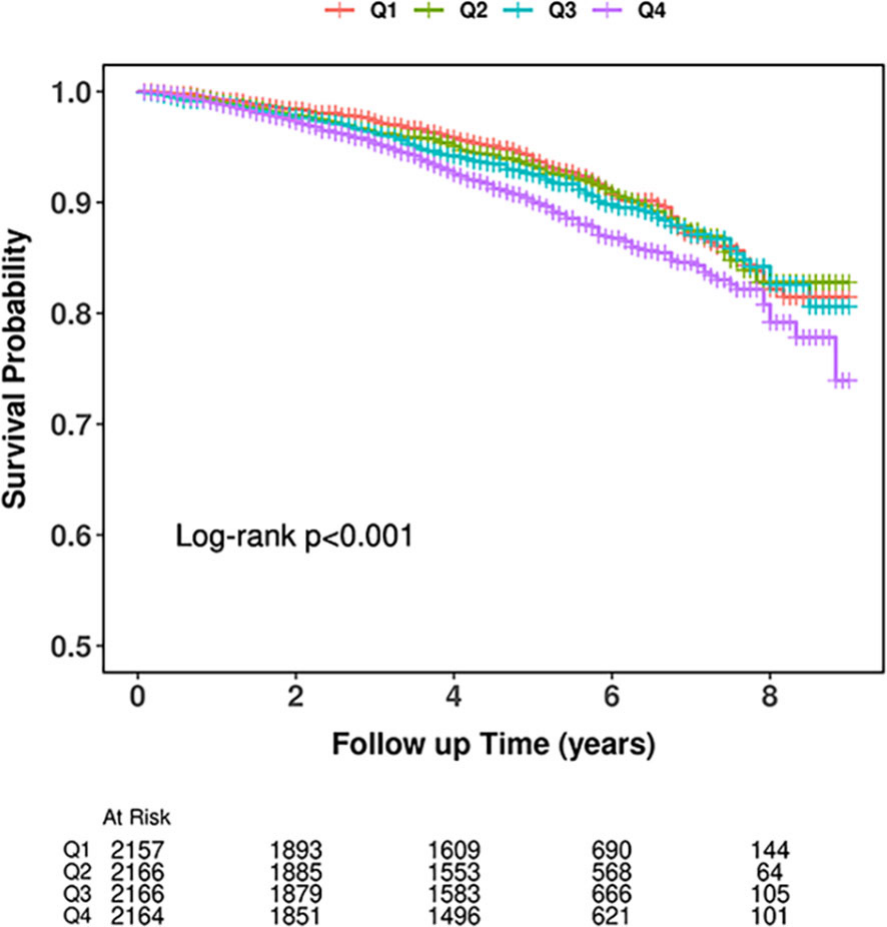
Kaplan-Meier event-free survival curve.

During a median follow-up of 5.2 years, 737 individuals developed CVD, comprising 442 cardiac events and 295 strokes. **Figure 2** presents the Kaplan-Meier curves for outcomes across PAC quartiles. These curves indicate a significantly elevated CVD risk among participants in the highest PAC quartile (P _log-rank_<0.001).

**Table 2** presents the association between baseline aldosterone levels and the risk of CVD. In the fully adjusted model, participants in the highest aldosterone quartile exhibited a 51% greater risk of CVD (HR = 1.51, 95% CI 1.23–1.86, P<0.001) compared with those in the lowest quartile. When analyzed as a continuous variable, each SD increase in aldosterone was associated with a 19% higher CVD risk (HR = 1.19, 95% CI 1.11–1.28, P<0.001).

**Table 2:** Association of PAC with risk of CVD.

PAC	Events (incidence rate*)	Crude model	Model 1	Model 2	Model 3
		HR (95%CI) P value	HR (95%CI) P value	HR (95%CI) P value	HR (95%CI) P value
Quartiles					
Quartile 1	168(15.7)	Reference	Reference	Reference	Reference
Quartile 2	159(15.7)	1.04(0.84-1.29) 0.731	1.08(0.87-1.34) 0.513	1.08(0.87-1.34) 0.488	1.08(0.87-1.35) 0.472
Quartile 3	182(17.6)	1.12(0.91-1.39) 0.278	1.16(0.94-1.43) 0.167	1.16(0.94-1.44) 0.168	1.17(0.95-1.45) 0.147
Quartile 4	228(22.4)	1.45(1.19-1.77) <0.001	1.54(1.26-1.88) <0.001	1.51(1.23-1.86) <0.001	1.51(1.23-1.86) <0.001
P for trend		<0.001	<0.001	<0.001	<0.001
Continuous (per SD) Threshold (14.22) ≤14.22 327 (15.7) >14.22 410 (19.9)		Reference 1.26 (1.09-1.46) 0.002	Reference 1.30 (1.12-1.50) <0.001	Reference 1.31 (1.13-1.50) <0.001	1.19(1.11-1.28) <0.001 Reference 1.31 (1.13-1.50) <0.001

*Incidence rates per 1000 person-years.

Model1: adjusted for age, gender, Duration of hypertension≥5 years, smoking status, alcohol use, DM at baseline; Model2: model 1+BMI, waist circumference, SBP, DBP, eGFR, BUN, TG, HDL-C, LDL-C, Lp(a), FBG, 24-h UNa, 24-h UK; Model3: model 2+antihypertensive agents, statins, antiplatelet agents, and antidiabetic drugs at discharge. BMI, body mass index; BUN, blood urea nitrogen; CI, confidence interval; DBP, diastolic blood pressure; eGFR, estimated glomerular filtration rate; FBG, fast blood glucose; HDL, high-density lipoprotein; HR, hazard ratio; LDL, low-density lipoprotein; PAC, plasma aldosterone concentration; SBP, systolic blood pressure; SD, standard deviation; TG, triglyceride; 24-h UNa, 24-h urinary sodium excretion; 24-h UK, 24-h urinary potassium excretion.

**Figure 3:**
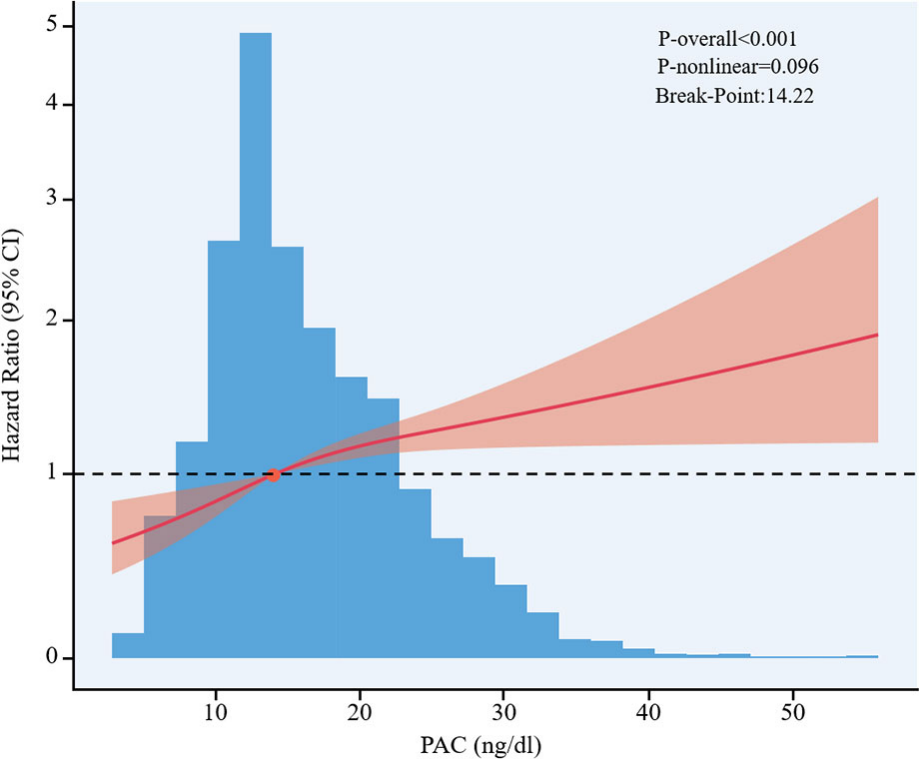
Dose-response association between PAC and CVD. Association between PAC and CVD were used a cox regression model of restricted cubic spline with 3 knots (at the 10th, 50th, and 90th percentiles) of PAC adjusting for potential covariates (including age, gender, Duration of hypertension≥5 years, smoking status, alcohol use, DM at baseline, body mass index, waist circumference, systolic blood pressure, diastolic blood pressure, eGFR [estimated glomerular filtration rate], BUN [blood urea nitrogen], triglyceride, HDL-C [high-density lipoprotein], LDL-C [low-density lipoprotein], Lp(a), fast blood glucose, 24-h UNa [24-h urinary sodium excretion], 24-h UK [24-h urinary potassium excretion], antihypertensive agents, statins, antiplatelet agents, and antidiabetic drugs at discharge.). CI, confidence interval; HR, hazard ratio; PAC, plasma aldosterone concentration.

When PAC was analyzed as a binary categorical variable based on its median value of 14.22 ng/dL, the upper median group exhibited a 1.31-fold increased risk of CVD compared to the lower median group (HR = 1.31, 95% CI 1.13–1.52, P < 0.001). Furthermore, the RCS analysis revealed a linear dose-response relationship between PAC and CVD, which became particularly pronounced once PAC exceeded 14.22 ng/dL, indicating a further escalation in CVD risk (P for nonlinear = 0.096, P for overall model < 0.001, **Figure 3**).

Sensitivity analyses that excluded participants with incident CVD within 12 months or suspected primary aldosteronism did not alter the observed association (**Supplementary Tables 2**, **3**).

The association between aldosterone levels and the incidence of cardiac events or stroke is presented in **Table 3**. Participants in the highest aldosterone quartile exhibited a 63% greater risk of cardiac events (HR = 1.62, 95% CI 1.24–2.12, P < 0.001) than those in the lowest quartile. Similarly, the highest quartile was associated with a 39% increased risk of stroke (HR = 1.39, 95% CI 1.01–1.92, P = 0.049) relative to the lowest quartile.

**Table 3:** Association of PAC with risk of cardiac events and stroke.

PAC	Events (incidence rate*)	Crude model	Model 1	Model 2	Model 3
		HR (95%CI) P value	HR (95%CI) P value	HR (95%CI) P value	HR (95%CI) P value
Cardiac events					
Quartiles					
Quartile 1	100(9.4)	Reference	Reference	Reference	Reference
Quartile 2	95(9.4)	1.05(0.79-1.39) 0.737	1.10(0.83-1.46) 0.496	1.10(0.83-1.46) 0.512	1.10(0.83-1.46) 0.499
Quartile 3	112(10.7)	1.16(0.89-1.53) 0.269	1.22(0.93-1.60) 0.150	1.24(0.94-1.62) 0.128	1.25(0.95-1.64) 0.112
Quartile 4	135(12.9)	1.44(1.12-1.87) 0.005	1.56(1.20-2.02) 0.001	1.61(1.23-2.10) 0.001	1.62(1.24-2.12) <0.001
P for trend		0.004	0.001	<0.001	<0.001
Continuous (per SD)		1.16(1.06-1.27) 0.001	1.21(1.10-1.32) <0.001	1.22(1.10-1.34) <0.001	1.22(1.11,1.34) <0.001
Stroke					
Quartiles					
Quartile 1	68(6.4)	Reference	Reference	Reference	Reference
Quartile 2	64(6.3)	1.02(0.73-1.44) 0.895	1.04(0.74-1.46) 0.832	1.04(0.74-1.46) 0.835	1.04(0.74-1.47) 0.822
Quartile 3	70(6.7)	1.06(0.76-1.48) 0.719	1.08(0.77-1.50) 0.668	1.07(0.76-1.49) 0.716	1.07(0.76-1.50) 0.713
Quartile 4	93(9.2)	1.45(1.06-1.98) 0.020	1.51(1.10-2.07) 0.010	1.39(1.01-1.93) 0.046	1.39(1.01-1.92) 0.049
P for trend		0.019	0.011	0.049	0.052
Continuous (per SD)		1.18(1.05-1.32) 0.004	1.20(1.07-1.35) 0.002	1.16(1.03-1.31) 0.014	1.16(1.03-1.31) 0.014

*Incidence rates per 1000 person-years.

Model1: adjusted for age, gender, Duration of hypertension≥5 years, smoking status, alcohol use, DM at baseline; Model2: model 1+BMI, waist circumference, SBP, DBP, eGFR, BUN, TG, HDL-C, LDL-C, Lp(a), FBG, 24-h UNa, 24-h UK; Model3: model 2+antihypertensive agents, statins, antiplatelet agents, and antidiabetic drugs at discharge. BMI, body mass index; BUN, blood urea nitrogen; CI, confidence interval; DBP, diastolic blood pressure; eGFR, estimated glomerular filtration rate; FBG, fast blood glucose; HDL, high-density lipoprotein; HR, hazard ratio; LDL, low-density lipoprotein; PAC, plasma aldosterone concentration; SBP, systolic blood pressure; SD, standard deviation; TG, triglyceride; 24-h UNa, 24-h urinary sodium excretion; 24-h UK, 24-h urinary potassium excretion.

### 3.3 Stratified analyses

**Figure 4:**
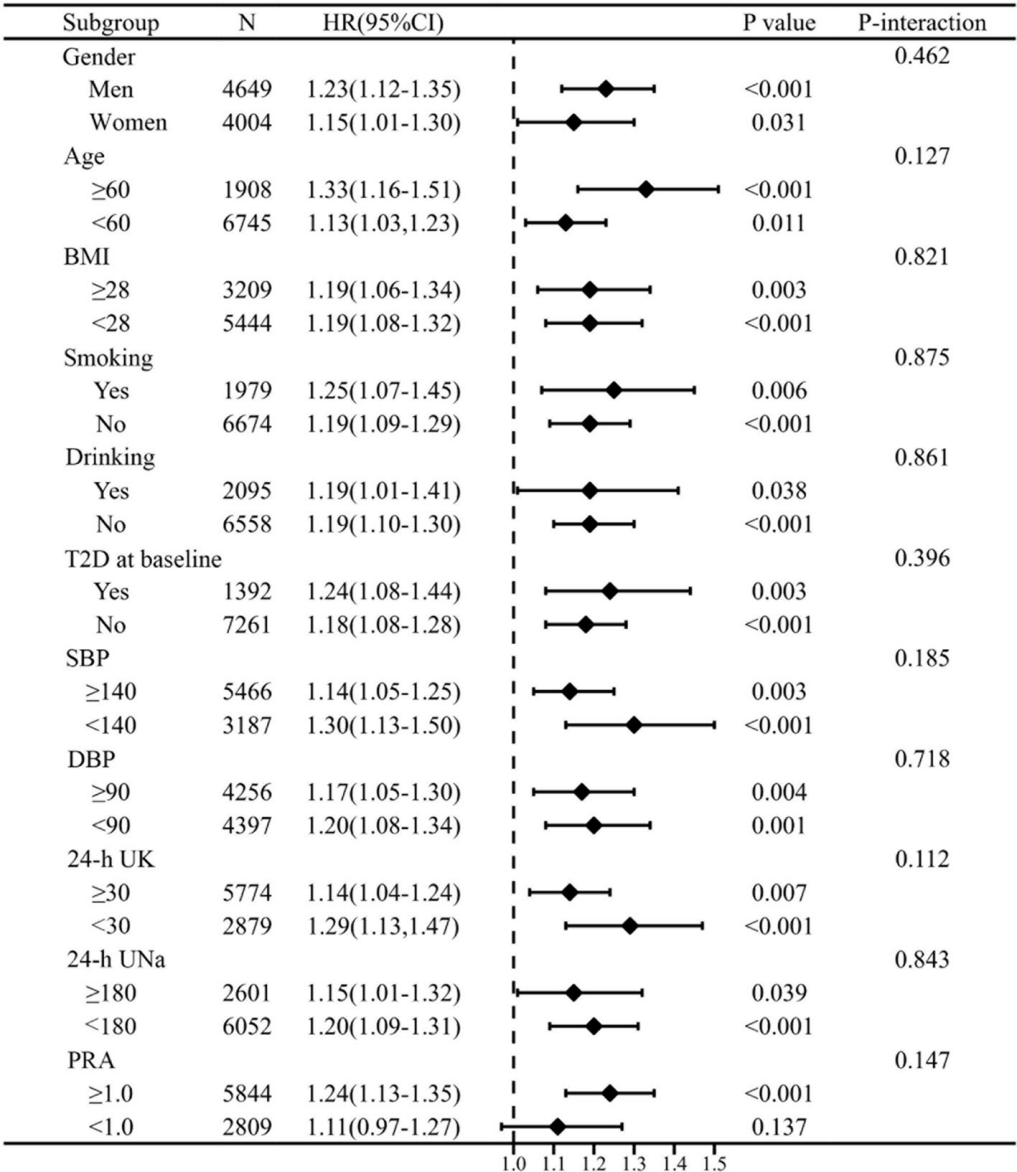
Stratification analyses on relationship of PAC with CVD. HRs werecalculated for per SD increase in PAC. Adjusted for age, gender, Duration ofhypertension≥5 years, smoking status, alcohol use, DM at baseline, BMI, waistcircumference, SBP, DBP, eGFR, BUN, triglyceride, HDL-C, LDL-C, Lp(a), fast blood glucose, 24-h UNa, 24-h UK, antihypertensive agents, statins, antiplatelet agents, and antidiabetic drugs at discharge. BMI, body mass index; BUN, blood urea nitrogen; CI, confidence interval; DBP, diastolic blood pressure; eGFR, estimated glomerular filtration rate; HDL, high-density lipoprotein; HR, hazard ratio; LDL, low-density lipoprotein; PAC, plasma aldosterone concentration; PRA, plasma renin activity; SBP, systolic blood pressure; SD, standard deviation; 24-h UNa, 24-h urinary sodium excretion; 24-h UK, 24-h urinary potassium excretion.

Stratified analyses evaluated the association between aldosterone, treated as a continuous variable per SD, and CVD risk across various subgroups (**Figure 4**). None of the examined variables—including gender, age, BMI, smoking or drinking status, baseline T2D, SBP, DBP, 24-h UNa, and 24-h UK—significantly modified the relationship between PAC and CVD (P-interaction > 0.05). In the subgroup with PRA < 1, higher PAC was also associated with increased CVD risk, although this association was not statistically significant.

## 4 Discussion

In this large-scale retrospective cohort, higher PAC was independently associated with an increased risk of CVD among hypertensive patients, even in the absence of primary aldosteronism. This association became more pronounced after excluding cases with suspected PA. These findings indicate that PAC could represent a target for CVD prevention in this patient population.

To the best of our knowledge, this is the first large-scale cohort study to investigate the association between aldosterone and CVD in hypertensive patients. Patients with PA are known to carry a significantly higher risk of CVD and mortality than those with essential hypertension, which is attributed to aldosterone excess and MR overactivation (7). Our findings align with several previous reports, thereby contributing new evidence to this area of ongoing debate and extending prior observations to a general hypertensive population. Specifically, higher PAC was associated with an elevated risk of CVD. Two recent studies further indicated that aldosterone was linked to all-cause mortality and adverse cardiac structural and functional changes only when PRA was suppressed (≤0.5 ng/mL per hour), suggesting that these associations may be partly attributable to overt or subclinical PA (25, 26). To minimize the influence of PA, we excluded patients with a definitive PA diagnosis. After also excluding those with suspected PA, the relationship between aldosterone and CVD became even stronger. These findings highlight the need to recognize the risk of target-organ damage resulting from elevated PAC across a broader hypertensive population. Moreover, given that aldosterone measurement is generally reserved only when PA is suspected, our findings are consistent with the 2024 ESC Guidelines for hypertension (29), which advocate routine aldosterone assessment in all hypertensive individuals to better prevent adverse cardiovascular outcomes.

Aldosterone contributes to the elevation of blood pressure and mediates cardiovascular injury through indirect mechanisms. Our findings demonstrate that aldosterone promotes cardiovascular disease independently of blood pressure and antihypertensive medications. Previous research has similarly established that aldosterone only partially mediates target organ damage through blood pressure elevation (25, 27), underscoring the significance of its direct effects on the cardiovascular system. *In vitro* studies demonstrate that aldosterone promotes remodeling and atherosclerosis by reducing nitric oxide production and enhancing collagen formation, fibrosis, and inflammation (30, 31). Aldosterone also induces cardiovascular damage by elevating systemic vascular resistance (32), impairing endothelial function (33), and increasing arterial stiffness (34). Given these mechanisms, MR antagonist treatment could offer therapeutic benefits. Animal models reveal that MR antagonists reduce vascular inflammation and fibrosis, protect vascular endothelial cells, and prevent adverse vascular remodeling (35, 36). However, the clinical use of MR antagonists remains limited. Current guidelines (37, 38) recommend these agents only for patients with acute myocardial infarction, heart failure with reduced ejection fraction, resistant hypertension, or primary aldosteronism, owing to side effects such as hyperkalemia and sex hormone-related effects. Recent results from the FIDELIO and FIGARO trials demonstrated that finerenone reduces the risk of chronic kidney disease progression and cardiovascular events in patients with chronic kidney disease and diabetes (39, 40). While these trials provide high-level evidence for the value of MR antagonists, our findings suggest that randomized controlled trials specifically in hypertensive patients may be necessary to evaluate their cardiovascular benefits.

This study has several strengths, including the evaluation of the relationship between PAC and cardiovascular events in a large-scale cohort of hypertensive patients with longitudinal follow-up. Our findings extend prior evidence to the broader hypertensive population, with implications for CVD prevention and treatment. We also accounted for the potential influence of antihypertensive agents, statins, antiplatelet drugs, and antidiabetic medications. Several limitations should nevertheless be acknowledged. Some patients did not complete the SIT, which may have led to the inclusion of individuals with PA. A sensitivity analysis that excluded suspected PA cases, however, yielded consistent results. Furthermore, the single-center design may limit the generalizability of our findings. Finally, as an observational cohort analysis, the study remains susceptible to unmeasured and residual confounding.

In summary, we demonstrated that higher PAC was associated with an increased risk of CVD in hypertensive patients, regardless of primary aldosteronism status. Our findings suggest that aldosterone should be measured in all hypertensive patients to better assess cardiovascular risk. Further investigation into MR antagonist therapy for CVD prevention in this population is therefore warranted.

## Data Availability

The raw data supporting the conclusions of this article will be made available by the authors, without undue reservation.

## Glossary

ACEI: angiotensin-converting-enzyme inhibitor
ARB: angiotensin receptor blockers
ARR: aldosterone to renin activity ratio
BMI: body mass index
CCB: calcium channel blocker
CVD: cardiovascular disease
DBP: diastolic blood pressure
eGFR: estimated glomerular filtration rate
HDL-C: high density lipoprotein cholesterol
HR: hazard ratio
LDL-C: low density lipoprotein cholesterol
MR: mineralocorticoid receptor
PA: primary aldosteronism
PAC: plasma aldosterone concentration
PRA: plasma renin activity
RCS: restricted cubic spline
SBP: systolic blood pressure
SD: standard deviation
SIT: saline infusion test.
