# Supplementary material for: Association between aldosterone and the risk of cardiovascular disease in hypertensive patients: a cohort study
Source: Front Endocrinol (Lausanne). 2026 Apr 23;17:1791569. doi: 10.3389/fendo.2026.1791569 (PMC13149068; doi:10.3389/fendo.2026.1791569)
Supplement: Supplementary file 1 [file DataSheet1.docx]

**SUPPLEMENTARY MATERIAL**

**Association between aldosterone and the risk of cardiovascular disease in hypertensive patients: a cohort study**

**All author’s names:** Lin Gan^1^, Nanfang Li^*2^, Mulalibieke Heizhati^2^, Hui Wang^2^, Ling Yao^2^, Li Cai^2^, Shasha Liu^2^, Jing Hong^2^, Deliang Zhang^2^

**Authors’ affiliations:**

^1^Graduate School, Xinjiang Medical University, Urumqi, China

^2^Hypertension Center of People’s Hospital of Xinjiang Uygur Autonomous Region, Xinjiang Hypertension Institute, NHC Key Laboratory of Hypertension Clinical Research, Key Laboratory of Xinjiang Uygur Autonomous Region “Hypertension Research Laboratory”, Xinjiang Clinical Medical Research Center for Hypertension (Cardio-Cerebrovascular) Diseases, Urumqi, China

**Corresponding author:** Professor Nanfang Li, Hypertension Center of People’s Hospital of Xinjiang Uygur Autonomous Region, 91 Tianchi Road, Urumqi, Xinjiang 830001, China.

E-mail: lnanfang2016@sina.com

**Supplementary Methods**

**The diagnostic criteria for secondary hypertension**

1) Renovascular hypertension: Renal artery stenosis was confirmed by angiogram and/or computed tomography angiography (CTA). Angiostenosis > 50% was confirmed positive ^[1]^.

2) Renal parenchymal hypertension: Patients were diagnosed with hypertension following a history of renal disease. These patients also exhibited abnormalities of urinary sediment including hematuria and proteinuria, and serum creatinine in the upright position (>177 umol/L) and/or pathological diagnosis after kidney biopsy and/or imaging showed abnormalities in morphology and structure of kidney.

3) Cushing’s syndrome: Cushing's syndrome was diagnosed through hormonal assessments, specifically via nonsuppressible serum cortisol following a 1 mg overnight dexamethasone test and a 48-hour low-dose (2 mg) dexamethasone suppression test. Localization of the source relied on ACTH level measurements, a 48-hour high-dose (8 mg) dexamethasone suppression test, and CT or MRI imaging. A cortisol level exceeding 5 µg/dL was defined as nonsuppressed, constituting a positive test result ^[2]^.

4) Pheochromocytoma: The diagnosis of pheochromocytoma relied on elevated plasma metanephrine and normetanephrine levels, with tumor localization achieved via CT, MRI, ¹³¹I-meta-iodobenzylguanidine scanning, and PET-CT ^[3]^. And final confirmation was made by pathological examination (adenoma adrenal medullary).

**Supplementary Tables**

| TableS1. Collinearity diagnostics | | | | | |
| --- | --- | --- | --- | --- | --- |
|  | All indicators | |  | Not including cholesterol | |
|  | Tolerance | VIF |  | Tolerance | VIF |
| Gender | 0.510 | 1.962 |  | 0.512 | 1.952 |
| Age | 0.364 | 2.748 |  | 0.365 | 2.742 |
| BMI | 0.322 | 3.140 |  | 0.323 | 3.100 |
| Waist circumference | 0.304 | 3.290 |  | 0.304 | 3.289 |
| Smoking status | 0.648 | 1.544 |  | 0.648 | 1.544 |
| Alcohol use | 0.636 | 1.572 |  | 0.636 | 1.572 |
| Hypertension Duration ≥5 | 0.851 | 1.175 |  | 0.851 | 1.175 |
| DM at baseline | 0.257 | 3.888 |  | 0.257 | 3.885 |
| Systolic blood pressure | 0.470 | 2.126 |  | 0.471 | 2.125 |
| Diastolic blood pressure | 0.404 | 2.476 |  | 0.404 | 2.473 |
| eGFR | 0.499 | 2.003 |  | 0.500 | 1.998 |
| Blood urea nitrogen | 0.818 | 1.223 |  | 0.818 | 1.223 |
| Fasting blood glucose | 0.588 | 1.701 |  | 0.589 | 1.698 |
| Total cholesterol | 0.082 | 12.217 |  |  |  |
| Triglyceride | 0.233 | 4.295 |  | 0.861 | 1.162 |
| HDL-C | 0.497 | 2.010 |  | 0.752 | 1.330 |
| LDL-C | 0.109 | 9.147 |  | 0.314 | 1.094 |
| 24-h UNa | 0.651 | 1.537 |  | 0.651 | 1.537 |
| 24-h UK | 0.697 | 1.434 |  | 0.697 | 1.434 |
| PRA | 0.913 | 1.096 |  | 0.913 | 1.095 |
| Medications at discharge |  |  |  |  |  |
| Antihypertensive agents≥2 | 0.806 | 1.241 |  | 0.806 | 1.241 |
| Statins | 0.591 | 1.693 |  | 0.591 | 1.693 |
| Antiplatelet agents | 0.627 | 1.596 |  | 0.627 | 1.595 |
| Antidiabetic drugs | 0.271 | 3.689 |  | 0.271 | 3.689 |
| BMI, body mass index;eGFR, estimated glomerular filtration rate; HDL-C, high density lipoprotein cholesterol; LDL-C, low density lipoprotein cholesterol; PRA, plasma renin activity; 24-h Una, 24-h urinary sodium excretion; 24-h UK, 24-h urinary potassium excretion. | | | | | |

| Table S2. Sensitivity analysis excluding subjects with incident CVD≤1 year (n=77). | | | | |  |
| --- | --- | --- | --- | --- | --- |
| PAC | Crude model | Model 1 | Model 2 | Model 3 |  |
|  | HR (95%CI) P value | HR (95%CI) P value | HR (95%CI) P value | HR (95%CI) P value |  |
| Quartiles |  |  |  |  |  |
| Quartile 1 | Reference | Reference | Reference | Reference |  |
| Quartile 2 | 1.03(0.82-1.29) 0.824 | 1.06(0.84-1.34) 0.608 | 1.06(0.84-1.33) 0.644 | 1.06(0.84-1.33) 0.628 |  |
| Quartile 3 | 1.08(0.86-1.35) 0.501 | 1.12(0.89-1.39) 0.336 | 1.11(0.89-1.40) 0.344 | 1.12(0.90-1.41) 0.311 |  |
| Quartile 4 | 1.45(1.18-1.79) 0.001 | 1.54(1.25-1.90) <0.001 | 1.48(1.19-1.84) <0.001 | 1.48(1.19-1.84) <0.001 |  |
| P for trend | <0.001 | <0.001 | <0.001 | <0.001 |  |
| Continuous (per SD) | 1.18(1.09-1.27) <0.001 | 1.21(1.13-1.31) <0.001 | 1.19(1.10-1.29) <0.001 | 1.19(1.10-1.29) <0.001 |  |
| Model1: adjusted for age, gender, Duration of hypertension≥5 years, smoking status, alcohol use, DM at baseline; Model2: model 1+BMI, waist circumference, SBP, DBP, eGFR, BUN, TG, HDL-C, LDL-C, Lp(a), FBG, 24-h UNa, 24-h UK; Model3: model 2+antihypertensive agents, statins, antiplatelet agents, and antidiabetic drugs at discharge. | | | | |  |
|  |  |  |  |  |  |
|  |  |  |  |  |  |

| Table S3. Sensitivity analysis excluding subjects with ARR≥20 and PAC≥12(n=1547). | | | | |  |
| --- | --- | --- | --- | --- | --- |
| PAC | Crude model | Model 1 | Model 2 | Model 3 |  |
|  | HR (95%CI) P value | HR (95%CI) P value | HR (95%CI) P value | HR (95%CI) P value |  |
| Quartiles |  |  |  |  |  |
| Quartile 1 | Reference | Reference | Reference | Reference |  |
| Quartile 2 | 1.15(0.90-1.46) 0.257 | 1.18(0.93-1.50) 0.166 | 1.16(0.91-1.48) 0.222 | 1.16(0.92-1.48) 0.217 |  |
| Quartile 3 | 1.24(0.98-1.57) 0.068 | 1.29(1.02-1.63) 0.035 | 1.26(0.99-1.60) 0.056 | 1.28(1.01-1.63) 0.043 |  |
| Quartile 4 | 1.46(1.16-1.82) 0.001 | 1.59(1.27-1.99) <0.001 | 1.50(1.18-1.91) 0.001 | 1.53(1.20-1.94) 0.001 |  |
| P for trend | 0.001 | <0.001 | 0.001 | 0.001 |  |
| Continuous (per SD) | 1.17(1.08-1.26) <0.001 | 1.21(1.12-1.31) <0.001 | 1.18(1.09-1.29) <0.001 | 1.19(1.10,1.30) <0.001 |  |
| Model1: adjusted for age, gender, Duration of hypertension≥5 years, smoking status, alcohol use, DM at baseline; Model2: model 1+BMI, waist circumference, SBP, DBP, eGFR, BUN, TG, HDL-C, LDL-C, Lp(a), FBG, 24-h UNa, 24-h UK; Model3: model 2+antihypertensive agents, statins, antiplatelet agents, and antidiabetic drugs at discharge. | | | | |  |
|  |  |  |  |  |  |
|  |  |  |  |  |  |

Reference

1. Baumgartner I, Lerman LO. Renovascular hypertension: screening and modern management. Eur Heart J. 2011 Jul;32(13):1590-8. doi: 10.1093/eurheartj/ehq510.
2. Guignat L, Bertherat J. The diagnosis of Cushing's syndrome: an Endocrine Society Clinical Practice Guideline: commentary from a European perspective. Eur J Endocrinol. 2010 Jul;163(1):9-13. doi: 10.1530/EJE-09-0627.
3. Lenders JW, Duh QY, Eisenhofer G, Gimenez-Roqueplo AP, Grebe SK, Murad MH, Naruse M, Pacak K, Young WF Jr; Endocrine Society. Pheochromocytoma and paraganglioma: an endocrine society clinical practice guideline. J Clin Endocrinol Metab. 2014 Jun;99(6):1915-42. doi: 10.1210/jc.2014-1498.
